# Fast Feature Extraction Method for Brillouin Scattering Spectrum of OPGW Optical Cable Based on BOTDR

**DOI:** 10.3390/s23198166

**Published:** 2023-09-29

**Authors:** Xiaojuan Chen, Haoyu Yu

**Affiliations:** School of Electronic Information Engineering, Changchun University of Science and Technology, Changchun 130022, China; yhaoyu0405@163.com

**Keywords:** brillouin scattering, image denoising, edge detection, distributed fiber optic sensing

## Abstract

Brillouin optical time domain reflectometry (BOTDR) detects fiber temperature and strain data and represents one of the most critical ways of identifying abnormal conditions such as ice coverage and lightning strikes on optical fiber composite overhead ground wire (OPGW) cable. Existing BOTDR extracts brillouin frequency shift (BFS) features with cumulative averaging and curve fitting. BFS feature extraction is slow for long-distance measurements, making realizing real-time measurements on fiber optic cables challenging. We propose a fast feature extraction method for block matching and 3D filtering (BM3D) + Sobel brillouin scattering spectroscopy (BGS). BM3D takes the advantage of non-local means (NLM) and wavelet denoising (WD) and utilizes the spatial-domain non-local principle to enhance the denoising in the transform domain. The global filtering capability of BM3D is utilized to filter out the low cumulative average BGS noise and the BFS feature extraction is completed using Sobel edge detection. Simulation verifies the feasibility of the algorithm, and the proposed method is embedded in BOTDR to measure 30 km of actual OPGW line. The experimental results show that under the same conditions, the processing time of this method is reduced by 37 times compared to that with the 50,000-time cumulative averaging + levenberg marquardt (LM) algorithm without severe distortion of the reference resolution. The method improves the sensor demodulation speed by using image processing technology without changing the existing hardware equipment, which is expected to be widely used in the new generation of BOTDR.

## 1. Introduction

China’s special optical fiber cables for power systems include OPGW, all-dielectric self-supporting (ADSS) optical fiber composition phase conductor (OPPC) cable, optical fiber composite low-voltage cable (OPLC), etc. [1]. OPGW cables are laid in the field with transmission lines and are susceptible to rain, snow, ice, and freezing weather [2]. Long-term ice-covered OPGW cable will cover arc prolapse and ice-covered dance, and severe ice coverage will lead to broken line falls, communication interruption, transmission line short circuits, and other faults. Ice is one of the main safety hazards of winter power operation and maintenance. The traditional OPGW cable condition detection technology is mainly based on point-type electronic sensors, which have problems such as power supply difficulties and the inability to distribute coverage monitoring [3]. Distributed optical fiber sensors (DOFSs), with the advantages of long detection distance, single-ended measurement, anti-electromagnetic interference, etc., can realize the fully distributed size of a variety of physical quantities along the fiber under test (FUT) [4]. Among them, BOTDR, based on the brillouin scattering principle, can realize the measurement of fiber temperature and strain changes and has been widely used in electric power communication, bridge construction, and building safety monitoring [5,6]. To realize the dynamic monitoring of temperature and stress changes in OPGW optical fiber cables, the fast and high-precision BFS extraction method has become a research hotspot.

The basic principle of BOTDR is to obtain the BFS distribution based on the BGS distribution to obtain the temperature and stress variation information of the FUT [7]. Since the BGS theoretically satisfies a Lorentzian-shaped distribution, the BFS is usually obtained with a curve fitting method (CFM). The accuracy of the CFM is affected by the signal-to-noise ratio (SNR) and the initial values of the relevant parameters [8]. When the SNR is low, an inappropriate initial value will directly affect the curve fitting accuracy, thus affecting the BFS extraction accuracy. In this regard, numerous scholars have proposed various methods for BFS extraction, such as the Levenberg–Marquardt algorithm for parameter optimization [9,10], the improved Newton algorithm based on a finite element analysis [11], and the cuckoo-optimized differential evolution (CS-IDE) algorithm [12]. While achieving better CFM accuracy, these algorithms require higher frequency sampling intervals and cumulative averaging. Cross-correlation, a principal component analysis, and support vector machines have also been shown to give better results [13,14,15,16]. Still, again, they are sensitive to the SNR and initial value of the signal, and the algorithm iterations are time-consuming and cannot satisfy dynamic monitoring.

To reduce the cumulative average number of times, in order to shorten the time consumption, experts and scholars have investigated the BGS denoising technique [17,18,19]. In 2012, Farahani et al. demonstrated a 90% reduction in cumulative averaging time using WD for denoising one-dimensional data [17]. In 2016, Soto, M. A. et al. proposed, for the first time, an image enhancement technique dealing with 2D BGS, which improved the sensor performance by a factor of 100 by using 2D NLM and WD to remove the noise [18]. This provided a new idea for BFS extraction. Deep learning has been widely used in image denoising [20]. In 2019, Cao, Z. et al. proposed a back propagation (BP) neural-network-based method for fiber frequency shift cross-section determination and BFS calculation [21]. In 2020, Chang, Y. et al. presented a distributed BFS-extracted convolutional neural network (BFSCNN) [22]. In 2022, Wu, H. et al. proposed a different image de-convolution technique based on 2D Wiener filtering to reduce the BGS blurring effect [23]. While deep learning algorithms have demonstrated superior performance in numerous tasks, the performance of models also needs to improve when data are insufficient or of poor quality and training duration and generalization are problematic [24].

In this work, we propose a BFS feature extraction method assisted with BM3D and Sobel, used to solve the problem of slow demodulation of BOTDR. For example, with 30 km of fiber to be tested, a sampling rate of 250 M/Sa, and 50,000 cumulative averaging processes, the time used for BFS demodulation is 2910 s. We consider the BGS as an image in which the roof-like edges are BFS. The global filtering capability of BM3D is used to filter out low-accumulated average BGS noise, and Sobel edge detection is used to complete BFS feature extraction to improve the performance of the BOTDR system. Simulations were conducted to verify the feature extraction capability of BM3D + Sobel and to analyze the filtering capability of BM3D for different noise-containing BGS images, the BFS feature extraction capability of the Sobel operator, and the processing speed of the algorithm. The proposed algorithm was experimentally validated on a 220 kV OPGW cable that had been in service for more than 10 years in a region of Jilin, China.

## 2. Materials and Methods

In this section, we propose the BM3D + Sobel method for rapid extraction of BFS data from the BOTDR sensor. Firstly, we treat the BGS data obtained from the BOTDR sensor as a noisy image. The BM3D algorithm is then applied to this image, primarily focusing on basic estimations and final estimations of the BGS image, resulting in a filtered BGS image [25,26]. Next, the filtered BGS image is convolved with the Sobel operator to obtain the BGS gradient image [27]. The sharpened feature image is obtained by subtracting the filtered BGS image from the BGS gradient image. Peak values in the feature image data are then identified, yielding the BFS matrix.

### 2.1. BOTDR BGS Image Denoising for BM3D

#### 2.1.1. Base Estimate

The BGS acquired with the BOTDR sensor is regarded as a noise-containing image. $I_{\mathrm{BGS}}$ is segmented into pixels of size N×N, and the reference block $Bx_{R}$ at a specific reference position $x_{R}$ is selected to match the similar partnership in the neighborhood. The Euclidean distance between $Bx_{i}$ and $Bx_{R}$ is used to determine its similarity to the reference block, and the Euclidean distance in the base estimation is calculated as follows:

(1) $$d_{\mathrm{noise}}\left(Bx_{R},Bx_{i}\right)=\frac{{\left\|\gamma^{\prime }\left(T_{2D}^{h}\left(Bx_{R}\right)\right)-\gamma^{\prime }\left(T_{2D}^{h}\left(Bx_{i}\right)\right)\right\|\;}_{2}^{2}}{{\left(N\right)}^{2}}$$

where $\gamma^{\prime }$ is hard threshold filtering and $T_{2D}^{h}$ is 2D linear filtering.

When the Euclidean distance between the similar block and the reference block is less than a fixed threshold, it is recognized as a similar block and vice versa as a non-similar block. The set of image blocks $S^{\mathrm{fth}}$ that are similar to the reference block is obtained and defined as

(2) $$S^{\mathrm{fth}}=\left\{Bx_{i}:d_{\mathrm{noise}}\left(Bx_{R},Bx_{i}\right)\leq \lambda_{1}^{\mathrm{fth}}\right\}$$

where $\lambda_{1}^{\mathrm{fth}}$ is the Euclidean distance threshold in the base estimation.

The similar image blocks are stacked together to form an $N\times N\times S^{\mathrm{fth}}$ array matrix $G^{\mathrm{fth}}$, and the resulting $G^{\mathrm{fth}}$ 3D matrix is co-filtered. The discrete cosine transform (DCT) is performed on the two-dimensional blocks in the three-dimensional matrix. The one-dimensional wavelet transform is performed on the third dimension in the three-dimensional matrix. Then, the estimation of the group of similar blocks of the image can be obtained with the three-dimensional inverse transform after the hard-threshold filtering:

(3) $${\hat{Q}}^{\mathrm{fth}}=Y_{3D}^{\mathrm{fth}-1}\left(\gamma \left(Y_{3D}^{\mathrm{fth}}\left(G^{\mathrm{fth}}\right)\right)\right)$$

where $Y_{3D}^{\mathrm{fth}}$ and $Y_{3D}^{\mathrm{fth}-1}$ denote the 3D transform and 3D inverse transform, respectively. The following is the hard threshold filter:

(4) $$\gamma \left(z\right)=\left\{\begin{array}{c} 0,\\ z, \end{array}\right.\begin{array}{cc} & \left|z\right|\leq \sigma \eta_{3D}\\ & \mathrm{otrherwise} \end{array}$$

where z is the matrix coefficient of the image block in the similarity cluster $S^{\mathrm{fth}}$, σ is the noise standard deviation, and $\eta_{3D}$ is the hard-threshold contraction coefficient.

During the image block matching process, multiple estimates may exist for the same pixel point in each matched block group, so a weighted average of each pixel point is required for aggregation. Assuming that a particular pixel x may occur within multiple pixel blocks, a weighted average of all image blocks containing pixel x is performed to obtain a basic estimate of the point, calculated as follows:

(5) $$I_{\mathrm{basic}}\left(x\right)=\frac{\sum_{x_{R}\in I}\sum_{x_{m}\in S^{\mathrm{fth}}}h_{\mathrm{fth}}{\hat{Q}}_{x_{R},x_{m}}^{\mathrm{fth}}}{\sum_{x_{R}\in I}\sum_{x_{m}\in S^{\mathrm{fth}}}h_{\mathrm{fth}}\delta_{x_{m}}\left(x\right)},\forall x\in I$$

(6) $$h_{\mathrm{fth}}=\left\{\begin{array}{ll} {\left(\sigma^{2}N_{h}\right)}^{-1}, & N_{h}\geq 1\\ 1, & 0 \end{array}\right.$$

where $I_{\mathrm{basic}}$ is the base estimation image, I is the set of image pixel blocks, x is the x image block, $S^{\mathrm{fth}}$ is the set of similar blocks of $Bx_{R}$, $x_{m}$ is the m image block of $S^{\mathrm{fth}}$, $x_{R}$ is the R image block of image I, $h_{\mathrm{fth}}$ is the magnitude of the weights of all non-zero elements in the matrix after the hard-threshold transformation, $\delta_{x_{m}}\left(x\right)$ is the characteristic function of the similar image blocks, ${\hat{Q}}_{x_{R},x_{m}}^{\mathrm{fth}}$ is the pixel estimation on the x image block in the set of similar blocks of the image at the base stage, $\sigma^{2}$ is the noise variance, and $N_{h}$ is the number of all non-zero elements in the matrix after the hard-threshold transformation.

#### 2.1.2. Final Estimate

The final estimation is similar to the grouping of the base estimation and results in two 3D matrices: $S^{\mathrm{final}}$ and $S^{\mathrm{fth}}$.

(7) $$S^{\mathrm{final}}=\left\{B^{\prime }x_{i}:d_{\mathrm{noise}}^{\mathrm{final}}\left(B^{\prime }x_{R},B^{\prime }x_{i}\right)\leq \lambda_{2}^{\mathrm{fth}}\right\}$$

(8) $$d_{\mathrm{noise}}^{\mathrm{final}}\left(B^{\prime }x_{R},B^{\prime }x_{i}\right)=\frac{{\left\|T_{2D}^{\mathrm{final}}\left(I_{\mathrm{basic}}\left(B^{\prime }x_{R}\right)\right)-T_{2D}^{\mathrm{final}}\left(I_{\mathrm{basic}}\left(B^{\prime }x_{i}\right)\right)\right\|}_{2}^{2}}{{\left(N_{1}\right)}^{2}}$$

Similarly, for collaborative filtering, the 3D matrix $S^{\mathrm{final}}$ of the base estimation result and the 3D matrix $S^{\mathrm{fth}}$ formed with the noisy image are subjected to the DCT 2D transform and 1D wavelet transform. $S^{\mathrm{final}}$ and $S^{\mathrm{fth}}$ are for joint Wiener filtering. Because the Wiener filter is a linear filter with the optimal mean square error, the filtering result can make the output image and the original noise-free signal with the minimum mean square error. Therefore, the use of Wiener filtering on the three-dimensional matrix for the coefficient contraction of the point-to-point can be achieved to restore the image details of the texture of the purpose. On top of this, the matrix is then inverted in three dimensions to obtain an estimate of the set of matching blocks:

(9) $${\hat{Q}}^{\mathrm{final}}=Y_{3D}^{\mathrm{final}-1}\left(H_{S^{\mathrm{final}}}^{\mathrm{final}}Y_{3D}^{\mathrm{final}}\left(G^{\mathrm{final}}\right)\right)$$

(10) $$H_{S^{\mathrm{final}}}^{\mathrm{final}}=\frac{{\left|T_{3D}^{\mathrm{final}}\left({\hat{Q}}_{\mathrm{final}}^{\mathrm{basic}}\right)\right|}^{2}}{{\left|T_{3D}^{\mathrm{final}}\left({\hat{Q}}_{\mathrm{final}}^{\mathrm{basic}}\right)\right|}^{2}+\sigma^{2}}$$

where $\left|\cdot \right|$ is the modulus of the complex numbers, $Y_{3D}^{\mathrm{final}}$ and $Y_{3D}^{\mathrm{final}-1}$ are the 3D linear transformation, $G^{\mathrm{final}}$ is the $N_{1}\times N_{1}\times S^{\mathrm{final}}$ 3D array matrix, $H_{S^{\mathrm{final}}}^{\mathrm{final}}$ is the Wiener filter shrinkage coefficients, ${\hat{Q}}_{\mathrm{final}}^{\mathrm{basic}}$ is the value of the 3D matrix at the base estimation stage, and $\sigma^{2}$ is the noise variance.

For the low-frequency components, the shrinkage factor is close to 1 because the similarity block makes the original energy of the image more concentrated, while the intensity of the noise is always the same. For the high-frequency components, the initial power of the image is minimal, so the shrinkage factor is close to 0 at this point. As for those intermediate frequency components, due to the existence of some higher frequency information in the image itself, there are some differences between similar blocks, so this part of the energy is lower. The difference with the noise is a little small. This time, though, the Wiener contraction can not only inhibit the function of the power of the noise but also retain the image as much as possible to preserve the details of the image information.

Like the base estimation, the 3D co-filtering needs to be aggregated after completion. Assuming that all pixels within a 3D combination are independent, the residual noise is proportional to the second-order paradigm of the contraction coefficient matrix, which defines the weights of the cross:

(11) $$h_{S^{\mathrm{final}}}^{\mathrm{final}}=\frac{1}{\sigma^{2}{\left\|H_{S^{\mathrm{final}}}^{\mathrm{final}}\right\|}_{2}^{2}}$$

Similarly, a Kaiser window is added to make the pixels in the center of the block have higher weights, thus reducing the boundary effect. The final estimated image can be represented as

(12) $$I_{\mathrm{final}}\left(x\right)=\frac{\sum_{x_{R}\in I}\sum_{x_{m}\in S^{\mathrm{final}}}h_{S^{\mathrm{final}}}^{\mathrm{final}}{\hat{Q}}_{x_{R},x_{m}}^{\mathrm{final}}}{\sum_{x_{R}\in I}\sum_{x_{m}\in S^{\mathrm{final}}}h_{S^{\mathrm{final}}}^{\mathrm{final}}\delta_{x_{m}}\left(x\right)},\forall x\in I$$

where $I_{\mathrm{final}}$ is the final estimated image, $S^{\mathrm{final}}$ is the set of similar blocks, and ${\hat{Q}}_{x_{R},x_{m}}^{\mathrm{final}}$ is the estimated value of pixels on the x image block in the collection of similar image blocks in the final estimation stage.

The BM3D algorithm achieves a good denoising effect through the base estimation and the enhanced image after the final estimation, especially the detailed texture in the image, which is well reproduced.

### 2.2. Sobel-Based BOTDR Brillouin Gain Spectrum Frequency Shift Feature Extraction

Edge detection techniques detect localized regions of significant change in an image [28], where image edges and contours are extracted by calculating the change in the image gradient. In the previous section, the BGS of the BOTDR sensing system is regarded as an image, and the most prominent edge in the figure is the location of the roof-like BFS. Therefore, the edge detection technique can extract the brillouin frequency shift features of the BGS image [29,30]. The convolution matrices for the X and Y directions are as follows:

(13) $$S_{x}=\left(\begin{array}{ccc} -1 & -2 & -1\\ 0 & 0 & 0\\ 1 & 2 & 1 \end{array}\right)$$

(14) $$S_{y}=\left(\begin{array}{ccc} -1 & 0 & 1\\ -2 & 0 & 2\\ -1 & 0 & 1 \end{array}\right)$$

BGS image edge gradient image G is

(15) $$G=\sqrt{G_{x}^{2}+G_{y}^{2}}$$

where $G_{x}=S_{x}\times I_{\mathrm{final}}$, $g_{x}$ is the gradient magnitude in the x direction of each pixel, and the same in the y direction, and $I_{\mathrm{final}}$ is the filtered BGS image.

The gradient direction θ of the image is as follows:

(16) $$\theta =\arctan\left(\frac{G_{y}}{G_{x}}\right)$$

To further sharpen the edge features of the BGS, we subtract image G from image $I_{\mathrm{final}}$, retaining only the data greater than 0, to obtain image $I_{\mathrm{BFS}\_G}$.

(17) $$I_{\mathrm{BFS}\_G}=\max\left\{I_{\mathrm{final}}-G,\;0\right\}$$

The BGS frequency shift position is determined, and the $I_{\mathrm{BFS}\_G}$ frequency peak matrix $I_{\mathrm{BFS}\_G}^{v\max}$ is extracted.

## 3. Numerical Simulation and Analysis

### 3.1. BM3D Filtering Performance Analysis

We simulated the brillouin interaction along a 50 km single-mode fiber. The full width at half maximum (FWHM) of the BGS was set to 32 MHz, and the scanning frequency range was 160 MHz in steps of 2 MHz. According to the definition of SNR, we could derive the following equation:

(18) $$\mathrm{SNR}=10\mathrm{lg}(\frac{\mathrm{sp}}{\mathrm{np}})$$

where sp is signal power and np is noise power.

Rearranging the equation, we obtained

(19) $$\mathrm{np}=\frac{\mathrm{sp}}{10^{\left(\frac{\mathrm{SNR}}{10}\right)}}$$

The sp could be estimated with the variance of the first row of the data matrix. Based on the given target SNR value and the variance of the data matrix, we could estimate the initial noise power, which was then used when generating variable SNR Gaussian white noise.

**Figure 1:**
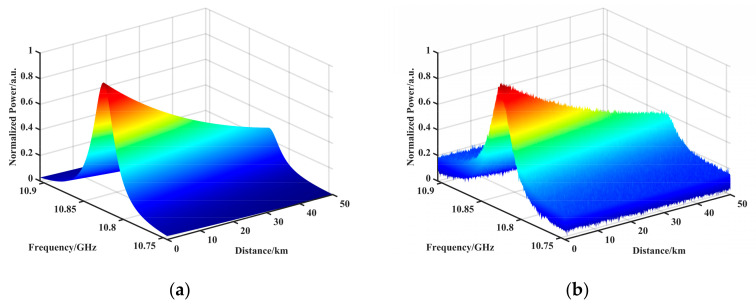
Simulation of BGS. (**a**) The ideal BGS. (**b**) The addition of a noisy BGS, where the starting SNR is 23.4 dB and the ending SNR is 9.75 dB.

The simulated BGS is shown in Figure 1, where (a) is the ideal BGS, and (b) is the addition of a noisy BGS, where the starting SNR is 23.4 dB and the ending SNR is 9.75 dB.

To quantitatively analyze the filtering effect of different algorithms on BGS, we simulated the low cumulative average number of times by corrupting the ideal BGS image with different noise levels. To verify the effect of BM3D filtering, BGS images with an SNR of 0.90 dB at the end of 50 km were filtered using the NLM, WD, Gaus, median, and mean algorithms. The image matrix size was 50,000 × 80 and the BM3D parameters were as follows: N=8, $\lambda_{1}^{\mathrm{fth}}=3000$, $N_{1}=8$, and $\lambda_{2}^{\mathrm{fth}}=400$. The NLM parameters were as follows: the similarity window was 3 × 3 and the search window was 13 × 13. The WD parameters were as follows: sym7 was used as the mother wavelet, the transform scale was 5, and hard thresholding was used for processing. The Gaus parameters were as follows: the standard deviation was 1 and the convolution kernel was 5 × 5. The mean parameter was as follows: the window size was 3 × 3. The median parameter was as follows: the window size was 3 × 3.

**Figure 2:**
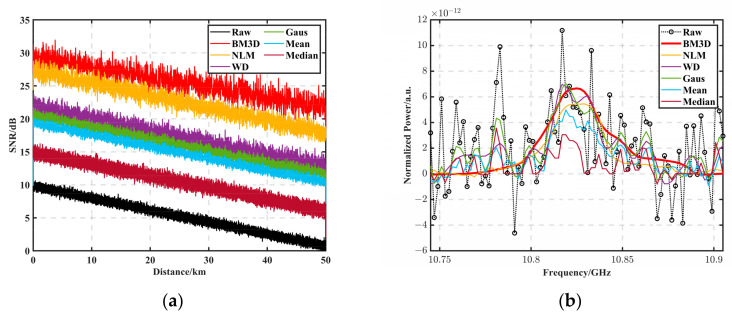
(**a**) Comparison of SNR improvement of different algorithms. (**b**) The effect before and after BGS filtering at 50 km; the black circle in the figure is the original BGS data, and the red solid line is the filtering processing result of the BM3D algorithm.

Figure 2 demonstrates the SNR enhancement of different algorithms with the same SNR, and the experimental results show that by applying the BM3D algorithm, the SNR of the original data obtained at 50 km with 0.90 dB could be dramatically improved to 21.89 dB, along with NLM to 17.86 dB, WD to 12.99 dB, Gaus to 11.71 dB, mean to 10.56 dB, and median to 6.19 dB. The BGS processed using the BM3D algorithm had a similar SNR to that of the cumulatively averaged 50,000-time BGS.

**Figure 3:**
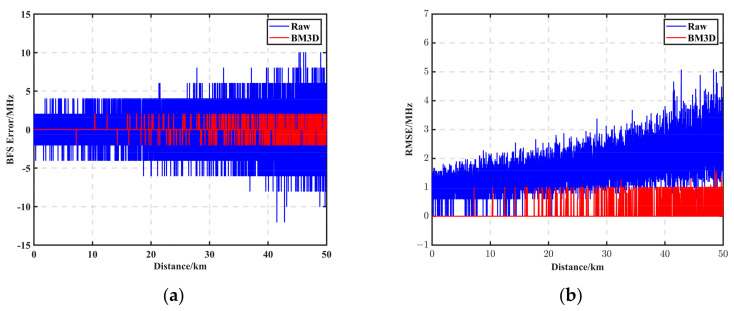
(**a**) BFS error in noise-containing data with an SNR of 0.90 dB at the end of 50 km versus the BM3D filtered data; the blue curve is the original data (BFS), and the red curve is the BM3D BFS curve. (**b**) RMSE in noise-containing data with an SNR of 0.90 dB at the end of 50 km versus the BM3D filtered data; the blue curve is the original data (RMSE), and the red curve is the BM3D RMSE curve.

**Figure 4:**
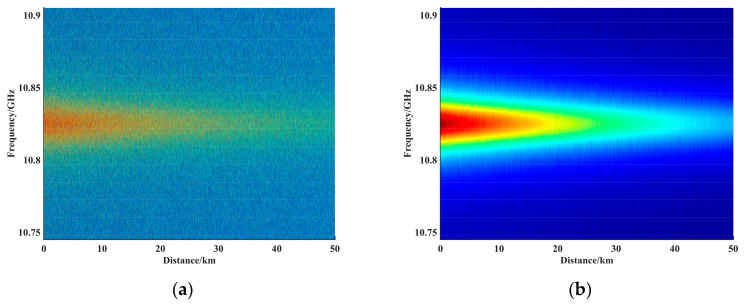
(**a**) Noisy BGS 2D view. (**b**) 2D view of BGS before and after filtering using the BM3D method.

To further study the filtering effect of the BM3D algorithm, Figure 2b shows the effect before and after the BGS filtering at 50 km, where the black circle is the original BGS data, and the red solid line is the filtering processing result of the BM3D algorithm. It can be seen that compared with other algorithms, the BM3D filtering effect was better, and the filtered curve was smoother and closer to the ideal BGS distribution. The BFS was obtained using the team’s previously published LSSVM curve fitting method [31]. Figure 3a demonstrates the BFS error between the noise-containing data with an SNR of 0.90 dB at the end of 50 km and the BFS error of the filtered data of the BM3D, with the blue curve being the BFS of the original data. The red curve is the BFS curve of the BM3D. The root mean square error (RMSE) was calculated for each unit of data using the BFS distribution of each of the ten sampling points in Figure 3a as the statistical unit. After BM3D filtering, the maximum error was reduced from 12 MHz to 2 MHz, the root mean square error (RMSE) at 1 m was reduced from 1.49 MHz to 0.0012 MHz, and the 50 km RMSE was reduced from 4.98 MHz to 0.86 MHz. Please refer to Table 1 for other algorithm comparisons. Figure 4a shows the 2D view of the noisy BGS, and Figure 4b shows the BGS 2D view before and after the filtering using the BM3D method.

### 3.2. Analysis of Spatial Resolution and Computational Complexity of BM3D Filtering

**Figure 5:**
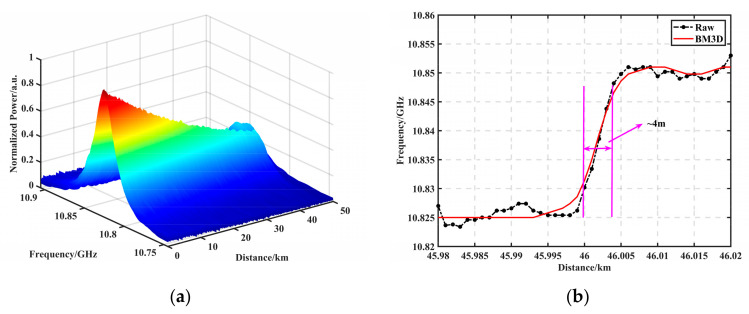
(**a**) BM3D-filtered 50-km-end 0.025-GHz-400-m-frequency-shift BGS 2D plot. (**b**) Frequency shift distribution plot at the end of the fiber, where the black dashed line is the raw data averaged over 50,000 times, and the reference spatial resolution was calculated to be 3.85 m. The red solid line is the curve at the end with an SNR of 0.90 dB filtered using the BM3D, and the spatial resolution was calculated to be 4 m.

We simulated a noise-containing BGS with an SNR of 0.90 dB at the end of 50 km, an initial frequency shift of 10.825 GHz, and a frequency shift of 0.025 GHz at the end of 400 m. The FWHM of the BGS was set to 32 MHz, and the scanning frequency range was 160 MHz in 2 MHz steps. Figure 5a shows the BM3D-filtered 50-km-end 0.025-GHz-400-m-frequency-shift BGS 2D plot, with the spatial resolution defined as the spatial length corresponding to 10% to 90% of the measured signal in the transition section and the BFS distribution obtained by averaging 50,000 times as the reference curve. Figure 5b shows the frequency shift distribution at the end of the fiber, where the black dashed line is the original data averaged over 50,000 times; the reference spatial resolution was calculated to be 3.85 m, and the red solid line is the curve after the SNR at the end was filtered with 0.90 dB using the BM3D filter. The spatial resolution was calculated to be 4 m, which does not produce a serious distortion of the reference resolution. The effect of different algorithms on the spatial resolution at the same SNR is discussed, and the specific results are shown in Table 1.

The following is a comparison of algorithm complexity: BM3D had higher computational complexity than other algorithms because BM3D combines information from both time and spatial domains. We used the processing speed of each algorithm as an evaluation index of its computational complexity, and we processed simulated BGS images with 50 km data points of 50,000 × 80 using MATLAB 2022b on computers configured with CPU i5-10210U and RAM 16G, respectively. The average processing times for BM3D, NLM, WD, Gaus, mean, and median were 243.22 s, 161.64 s, 6.75 s, 5.31 s, 4.98 s, and 4.14 s, respectively, for the same SNR. MATLAB was used to process the above data using MATLAB 2022b in a computer with a CPU i9-12900K and 128G RAM. The average time for BM3D, NLM, WD, Gaus, mean, and median processing was 63.83 s, 46.18 s, 2.12 s, 1.46 s, 1.52 s, and 1.11 s, respectively, which represented an average of 3.3-times-faster processing speed. Table 1 demonstrates the filtering effect and processing time of different filtering algorithms with different SNR, from which it can be seen that the BM3D algorithm outperformed the other algorithms in terms of SNR improvement and BFS error improvement. The experimental results show that the BM3D processing time was long, but the processing speed could be improved by using a better-configured computer.

### 3.3. Sobel Feature Extraction Performance Analysis

**Figure 6:**
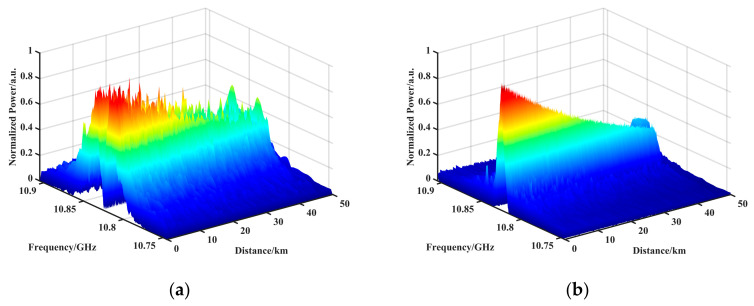
(**a**) Gradient map after convolution. (**b**) BGS image after further sharpening.

We simulated brillouin interactions along a 50 km single-mode fiber. The FWHM of the BGS was set to 32 MHz, the scanning frequency range was 160 MHz, the step size was 2 MHz, the SNR at the end was 0.90 dB, the frequency shift information was set to 46–50 km at the frequency shift, the frequency offset was 0.025 GHz, and the size of the resulting BGS data matrix was 50,000 × 80. The BGS data matrix was first globally denoised using the BM3D analyzed in the previous section, and then the filtered BGS image matrix was convolved using the Sobel operator. Figure 6a shows the gradient map after convolution, in which it can be seen that the gradient on both sides of the location where the BFS is located is much larger than the gradient in the middle, which proves that the BFS in the BGS had edge properties. Figure 6b shows the BGS map after further sharpening. Using the pre-convolution BGS data to subtract the post-convolution gradient data and retaining the data greater than zero for normalization, it was possible to further highlight the BFS part.

**Figure 7:**
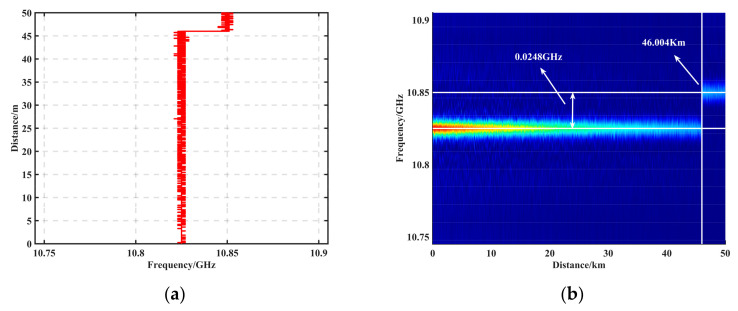
(**a**) BFS plot after peak feature extraction after Sobel operator processing. (**b**) BGS 2D view after Sobel processing.

To verify the accuracy and processing speed of the algorithm, the filtered BGS was subjected to BFS feature extraction using Sobel, LM, and LSSVM algorithms, respectively. LM and LSSVM are curve-fitting methods. Among them, LM is an experimental method for commercially available products. Figure 7a shows the BFS plot after peak feature extraction after Sobel operator processing, and Figure 7b shows the BGS 2D view after Sobel processing. The BFS was calculated to be frequency shifted from 10.825 GHz to 10.8448 at 46.004 km, with an offset of 0.0248 GHz and an offset error of 0.2 MHz. Table 2 demonstrates the extraction accuracy and processing speed of each algorithm (CPU i9-12900K, RAM 128G MATLAB 2022b), where the frequency shift of each interval was determined based on the average value, so the average error of each interval segment evaluated the BFS feature extraction accuracy. As shown in Table 2, the Sobel algorithm improved the measurement accuracy by up to 16 times and the processing speed by up to 314 times.

## 4. Demonstration Experiment

**Figure 8:**
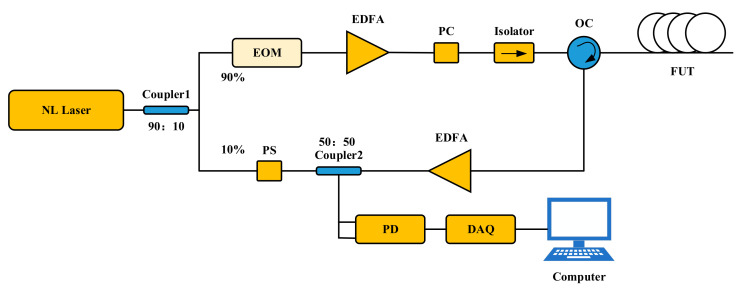
BOTDR sensing system.

We built the BOTDR sensing system as shown in Figure 8. A narrow linewidth laser with a center wavelength of 1550.12 nm and a linewidth of 100 kHz was used. The light was divided into two paths of 90% and 10% with coupler1, of which 90% of the light was the detection light. The light signal was modulated with an electro-optical modulator (EOM), amplified with an erbium-doped fiber amplifier (EDFA), passed through a polarizer controller (PC), and then transmitted to the fiber optic transmission unit (FUT) via an optical isolator (Isolator) and an optical circulator (OC). Ten percent of the light served as the reference light. The scattered light, which was adjusted with a polarization scrambler (PS) and amplified with the EDFA, was coupled into the dual-input photo detector (PD) through a 50/50 coupler2, where it was converted into electrical signals. Then, it was sent to the computer for the BFS extraction through the data acquisition card (DAQ).

**Figure 9:**
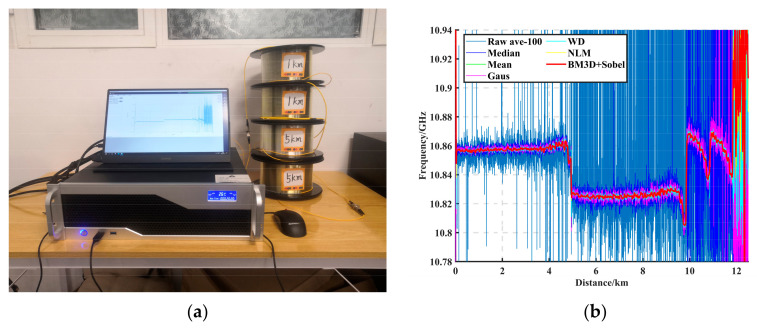
(**a**) Physical diagram of the experimental system. (**b**) Effect of BFS feature extraction for each algorithm.

We link the BOTDR with 12 km (5 km + 5 km + 1 km + 1 km) single-mode fiber, and Figure 9a shows the physical diagram of the experimental system. The BOTDR parameters were set to a detection distance of 12 km, with the scan frequency range set to 160 MHz in 2 MHz steps, the spatial resolution set to 5 m, and the cumulative average set to 100 times (ave-100). The BGS data obtained with cumulative ave-100 were used as the original data, and the BGS data obtained with cumulative ave-80,000 were used as the reference data. Figure 9b shows the effect of BFS feature extraction for each algorithm, from which it can be seen that BM3D + Sobel BFS error was minimized. Table 3 is the comparison of system performance improvement by different filtering algorithms. Compared with ave-100, SNR was improved by 20.29 dB, RMSE was reduced from 119.04 MHz to 0.12 MHz, and spatial resolution was improved by 3 m. Compared with ave-50,000, SNR was improved by 1.91 dB, RMSE was reduced from 2.26 MHz to 0.12 MHz, spatial resolution was improved by 0.5 m, and calculation time was shortened by 36.9 times.

**Figure 10:**
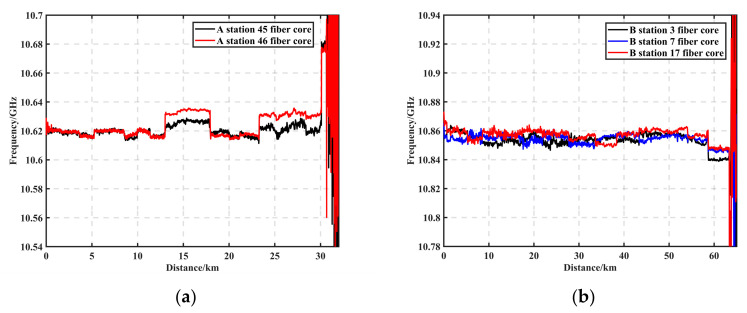
(**a**) Thirty-kilometer BFS curves measured with BM3D + Sobel. (**b**) Sixty-two-kilometer BFS curves measured with BM3D + Sobel.

Experimental validation was carried out for 220 kv OPGW fiber optic cables that had been in service for more than 10 years during January 2023 in a region of Jilin, China. The BOTDR device was placed at station A, with the scan frequency range set to 160 MHz in 2 MHz steps, the cumulative average set to 100 times, and spatial resolution set to 5 m. Figure 10a shows the 30 km BFS curves measured using BM3D + Sobel, from which it can be seen that the length of the line was about 30 km. The difference in BFS between 45-core and 46-core in the figure is due to different fiber types or different batches of fibers. This difference can be used to analyze the temperature or strain anomalies to analyze the fiber optic cable status if there is an abnormal location of the BFS. The measured BGS data matrix was 62,458 × 80, and the device’s hardware configuration was CPU i9-12900K and RAM 128G. Taking the ave-80,000 + LM as the reference measurement, the processing speed and accuracy of the cumulative ave-50,000 + LM and BM3D + Sobel algorithms were compared, respectively, in which the cumulative ave-50,000 + LM processing time was 2904 s, the 30 km RMSE was 3.62 MHz, and the spatial resolution was 8 m. The processing time of the BM3D + Sobel algorithm was 78.59 s, the 30 km RMSE was 0.27 MHz, and the spatial resolution was 4 m, which meant the processing time was reduced by a factor of 37 and the average error by a factor of 14.

To further validate the reproducibility of the method proposed in this paper, maintain the same hardware conditions and calibration methods. The BOTDR device was placed at station B to detect cores 3, 7, and 17, with the scan frequency range set to 160 MHz in 2 MHz steps, the cumulative average set to 100 times, and spatial resolution set to 10 m. Figure 10b shows the 62 km BFS curves measured using BM3D + Sobel. The measured BGS data matrix was 127,043 × 80, ave-50,000 + LM processing time was 5793 s, 62 km RMSE was 6.81 MHz, BM3D+Sobel algorithm was 163.42 s, and 62 km RMSE was 0.83 MHz. The speed and accuracy improvements remain in the same order of magnitude as in the previous section.

## 5. Conclusions

In this study, since the cumulative averaging number and BFS curve fitting are important determinants of dynamic sensing speed, we propose and demonstrate the use of the BM3D + Sobel algorithm to enhance the speed and accuracy of brillouin scattering spectrum feature extraction. The BM3D algorithm was utilized instead of cumulative averaging, and the Sobel edge detection algorithm was utilized instead of curve fitting. The global filtering capability of BM3D was verified with simulation. The simulation results show that, among the six filtering algorithms, NLM, WD, Gaus, median, mean, and BM3D, there was an average improvement of 20.34 dB (min, 19.35 dB; max, 20.99 dB) in the BM3D SNR, and the RMSE was reduced by up to 4 MHz under different degrees of noise-corrupted BGS images without serious distortion of the reference resolution. The Sobel edge detection algorithm improved the BFS feature extraction accuracy by up to 16 times, with an offset error of 0.2 MHz, and the processing speed by up to 314 times, with a time of 0.2 s. Under the 12 km laboratory measurement condition, compared with ave-100, SNR was improved by 20.29 dB, RMSE was reduced from 119.04 MHz to 0.12 MHz, and spatial resolution was improved by 3 m. Under the 30 km real measurement condition, the cumulative ave-50,000 + LM algorithm took 2904 s to process, the 30 km RMSE was 3.62 MHz, and the spatial resolution was 8 m. The BM3D + Sobel algorithm took 78.59 s to process, the 30 km RMSE was 0.27 MHz, and the spatial resolution was 4 m, which was a 37-fold shortening of the processing time. For the same CPU, although BM3D+Sobel was slower than NLM and WD methods, it still has advantages in measurement accuracy and spatial resolution.

In addition, compared with the deep learning network, the BM3D + Sobel algorithm had lower computational complexity, did not depend on the training dataset, and could accomplish faster BFS feature extraction without changing the existing hardware conditions, which is suitable for any BOTDR. This provides a technical solution for BOTDR to realize real-time measurements in the power system.

## Figures and Tables

**Table 1:** Performance comparison of different SNR filtering algorithms.

Algorithm	SNR of Raw Data/dB	Improvement in SNR/dB	Reduction in RMSE/MHz	Spatial Resolution/m	Fiber Length/km	i5 16G Processing Time/s	i9 128G Processing Time/s
BM3D	0.90	20.99	4.00	4	50	243.22	63.83
	3.67	20.70	2.74				
	6.77	20.34	2.68				
	9.75	19.35	1.54				
NLM	0.90	16.96	3.49	8	50	161.64	46.18
	3.67	16.42	2.22				
	6.77	15.72	1.40				
	9.75	15.03	0.57				
WD	0.90	12.09	3.10	12	50	6.75	2.12
	3.67	12.15	1.31				
	6.77	12.05	1.19				
	9.75	12.10	0.52				
Gaus	0.90	10.81	2.33	15	50	5.31	1.46
	3.67	10.85	1.18				
	6.77	10.70	1.12				
	9.75	10.82	0.31				
Mean	0.90	9.66	1.99	15	50	4.98	1.52
	3.67	9.67	0.99				
	6.77	9.65	1.02				
	9.75	9.64	0.26				
Median	0.90	5.29	1.04	15	50	4.14	1.11
	3.67	5.27	0.85				
	6.77	5.07	0.59				
	9.75	5.03	0.12				

**Table 2:** Extraction accuracy and processing speed of each algorithm.

Algorithm	Average Error/MHz	Processing Time/s
LM	3.2	351.81
LSSVM	2	229.55
Sobel	0.2	1.12

**Table 3:** Comparison of system performance improvement by different filtering algorithms.

Algorithm	12 km SNR/dB	12 km RMSE/MHz	Spatial Resolution/m	Processing Time/s
ave-100	1.07	119.04	8	2.41
ave-50,000	19.45	2.26	5	618.74
BM3D + Sobel	21.36	0.12	4.5	16.75
NLM	17.41	0.64	8	12.13
WD	13.05	0.91	10	1.34
Gaus	10.86	2.25	15	1.01
Mean	9.84	2.97	15	0.95
Median	6.32	3.11	15	0.89

## Data Availability

The data presented in this study are available on request from the corresponding author. The data are not publicly available due to potential commercial values.

## References

[1] Lin R., Zhu Y., Tian L., Zhou L., Liu W., Cheng L. (2021). On-situ monitoring of sleet-thawing for OPGW based on long distance BOTDR. Optoelectron. Lett..

[2] Cai L., Han T., Wang J., Xue J., Xu F., Zhou M., Fan Y. (2020). Experimental analysis of the strands breaking characteristics of optical fibre composite overhead ground wire due to simulating lightning strike. IET Gener. Transm. Distrib..

[3] Sun J., Zhang Z., Li Y., Yan Z., Zhai T., Li L., Xiao Z. (2021). Distributed Transmission Line Ice-Coating Recognition System Based on BOTDR Temperature Monitoring. J. Light. Technol..

[4] Dong Y. (2021). High-Performance Distributed Brillouin Optical Fiber Sensing. Photonic Sens..

[5] Nie T., Li J., Ding Y., Zhang Z., Li B., Dong W. (2021). Fast extraction for Brillouin frequency shift in BOTDA system. Opt. Quantum Electron..

[6] Gao L., Han C., Xu Z., Jin Y., Yan J. (2019). Experimental Study on Deformation Monitoring of Bored Pile Based on BOTDR. Appl. Sci..

[7] Soto M.A., Thévenaz L. (2013). Modeling and evaluating the performance of Brillouin distributed optical fiber sensors. Opt. Express.

[8] Bado M.F., Casas J.R. (2021). A review of recent distributed optical fiber sensors applications for civil engineering structural health monitoring. Sensors.

[9] Zhang Y., Fu G., Liu Y., Bi W., Li D. (2013). A novel fitting algorithm for Brillouin scattering spectrum of distributed sensing systems based on RBFN networks. Opt.—Int. J. Light Electron Opt..

[10] Zhang Y., Yu C., Fu X., Li D., Jia W., Bi W. (2014). An improved Newton algorithm based on finite element analysis for extracting the Brillouin scattering spectrum features. Measurement.

[11] Zhao L., Li Y., Xu Z. (2014). A fast and high accurate initial values obtainment method for Brillouin scattering spectrum parameter estimation. Sens. Actuators A Phys..

[12] Zhang Y., Yu C., Fu X., Liu W., Bi W. (2015). Spectrum parameter estimation in Brillouin scattering distributed temperature sensor based on cuckoo search algorithm combined with the improved differential evolution algorithm. Opt. Commun..

[13] Farahani M.A., Castillo-Guerra E., Colpitts B.G. (2013). A Detailed Evaluation of the Correlation-Based Method Used for Estimation of the Brillouin Frequency Shift in BOTDA Sensors. IEEE Sens. J..

[14] Azad A.K., Khan F.N., Alarashi W.H., Guo N., Lau A.P.T., Lu C. (2017). Temperature extraction in Brillouin optical time-domain analysis sensors using principal component analysis based pattern recognition. Opt. Express.

[15] Wu H., Wang L., Guo N., Shu C., Lu C. (2017). Brillouin Optical Time-Domain Analyzer Assisted by Support Vector Machine for Ultrafast Temperature Extraction. J. Light. Technol..

[16] Zhu H., Yu L., Zhang Y., Cheng L., Zhu Z., Song J., Zhang J., Luo B., Yang K. (2020). Optimized Support Vector Machine Assisted BOTDA for Temperature Extraction with Accuracy Enhancement. IEEE Photonics J..

[17] Farahani M.A., Wylie M.T.V., Castillo-Guerra E., Colpitts B.G. (2013). Reduction in the Number of Averages Required in BOTDA Sensors Using Wavelet Denoising Techniques. J. Light. Technol..

[18] Soto M.A., Ramírez J.A., Thévenaz L. (2016). Intensifying the response of distributed optical fibre sensors using 2D and 3D image restoration. Nat. Commun..

[19] Qian X., Jia X., Wang Z., Zhang B., Xue N., Sun W., He Q., Wu H. (2017). Noise level estimation of BOTDA for optimal non-local means denoising. Appl. Opt..

[20] Tian C., Fei L., Zheng W., Xu Y., Zuo W., Lin W. (2020). Deep learning on image denoising: An overview. Neural Netw..

[21] Cao Z., Guo N., Li M., Yu K., Gao K. (2019). Back propagation neutral network based signal acquisition for Brillouin distributed optical fiber sensors. Opt. Express.

[22] Chang Y., Wu H., Zhao C., Shen L., Fu S., Tang M. (2020). Distributed Brillouin frequency shift extraction via a convolutional neural network. Photonics Res..

[23] Wu H., Guo N., Feng D., Yin G., Zhu T. (2022). Enhancing Spatial Resolution of BOTDR Sensors using Image Deconvolution. Opt. Express.

[24] Li B., Jiang N., Han X. (2023). Denoising of BOTDR Dynamic Strain Measurement Using Convolutional Neural Networks. Sensors.

[25] Abubakar A., Zhao X., Li S., Takruri M., Bastaki E., Bermak A. (2018). A Block-Matching and 3-D Filtering Algorithm for Gaussian Noise in DoFP Polarization Images. IEEE Sens. J..

[26] Wu H., Wang L., Zhao Z., Guo N., Shu C., Lu C. (2018). Brillouin optical time domain analyzer sensors assisted by advanced image denoising techniques. Opt. Express.

[27] Tian R., Sun G., Liu X., Zheng B. (2021). Sobel Edge Detection Based on Weighted Nuclear Norm Minimization Image Denoising. Electronics.

[28] Elharrouss O., Hmamouche Y., Idrissi A.K., El Khamlichi B., El Fallah-Seghrouchni A. (2023). Refined edge detection with cascaded and high-resolution convolutional network. Pattern Recognit..

[29] Li J., Zhou W., Zhang Y., Dong W., Zhang X. (2019). A Novel Method of the Brillouin Gain Spectrum Recognition Using Enhanced Sobel Operators Based on BOTDA System. IEEE Sens. J..

[30] Li X., Chang Q., Li Y., Miyazaki J. (2022). Multi-Directional Sobel Operator Kernel on Gpus. SSRN Electron. J..

[31] Chen X., Yu H., Huang W. A high accurate fitting algorithm for Brillouin scattering spectrum of distributed sensing systems based on LSSVM networks. Proceedings of the 2021 International Conference on Electronic Information Engineering and Computer Science (EIECS).

